# Effects of acupuncture on the pregnancy outcomes of frozen-thawed embryo transfer: A systematic review and meta-analysis

**DOI:** 10.3389/fpubh.2022.987276

**Published:** 2022-09-09

**Authors:** Can Zhu, Wanting Xia, Jinzhu Huang, Xuan Zhang, Fangyuan Li, Xiaorun Yu, Jiamin Ma, Qian Zeng

**Affiliations:** ^1^Department of Gynecology, Hospital of Chengdu University of Traditional Chinese Medicine, Chengdu, China; ^2^School of Basic Medical Sciences, Guizhou University of Traditional Chinese Medicine, Guiyang, China; ^3^School of Nursing, Chengdu University of Traditional Chinese Medicine, Chengdu, China

**Keywords:** infertility, acupuncture, frozen-thawed embryo transfer (FET), pregnancy outcomes, endometrial morphology, systematic review and meta-analysis

## Abstract

**Background:**

Acupuncture is increasingly used as adjuvant therapy for infertile women undergoing frozen-thawed embryo transfer (FET); however, its effects and safety are highly controversial. This study aimed to evaluate the pooled effects of adjuvant acupuncture on FET pregnancy outcomes.

**Methods:**

We considered only randomized controlled trials (RCTs) that compared acupuncture with sham acupuncture or no adjuvant treatment during FET and the primary outcome was clinical pregnancy rate. Two authors separately selected studies, extracted data, and performed a risk of bias assessment. Pooled data were expressed as risk ratio (*RR*) or mean difference (*MD*), with a 95% confidence interval (*CI*). In addition, we conducted subgroup and sensitivity analyses to investigate the sources of heterogeneity, and we also constructed funnel plots to assess the likelihood of publication bias. Finally, Grading of Recommendation, Assessment, Development, and Evaluation (GRADE) was applied to evaluate the quality of evidence.

**Results:**

A total of 14 RCTs with a total of 1,130 participants were included in the study. We found significant effects of acupuncture adjuvant to FET on the outcomes of clinical pregnancy rate (*RR* = 1.54, 95% *CI* [1.28, 1.85], *I*^2^ = 34%; 14 trials), biochemical pregnancy rate (*RR* = 1.51, 95% *CI* [1.21, 1.89]; 5 trials), endometrial thickness (*MD* = 0.97, 95% *CI* [0.43, 1.51]; 12 trials), and endometrial pattern (*RR* = 1.41, 95% *CI* [1.13, 1.75]; 7 trials). For live birth rate (*RR* = 1.48, 95% *CI* [0.90, 2.43], 4 trials), there were no statistical effectiveness. For subgroup analyses, most variables had tolerable heterogeneity (*I*^2^ = 0%) except for trials that were sham-controlled, performed acupuncture only after FET, or <5 times, which appeared to interpret most of the heterogeneity. Additionally, the quality of evidence of all outcomes in this review ranged from low to moderate.

**Conclusion:**

Acupuncture could be instrumental in the pregnancy outcomes of FET, and has very few risks of severe adverse events; however, the quality of evidence is unsatisfactory. Further research with rigorous methodological quality should be considered, and the protocols of acupuncture also need more investigations (e.g., appropriate control groups, sessions, and times).

## Introduction

Infertility has become commonplace in recent years, with average morbidity of 8–12% worldwide (1) and 25% in China (2), resulting in severe influences on countless families and public health. Frozen-thawed embryo transfer (FET), as a crucial part of the assisted reproductive technique, is frequently performed for infertile women, especially when they suffer from cycle cancellation or implantation failure in previous embryo transfer cycles and have at least one eligible frozen embryo (3). Increasing evidence has shown that FET significantly decreases the risk of ovarian hyperstimulation syndrome (4), and frozen single blastocyst transfer has a higher live birth rate than does fresh cycle in ovulatory women (5). Nevertheless, despite advances in reproductive techniques, the clinical pregnancy rate of FET remains low, at only 58.7% (6).

Therefore, investigating adjuvant therapies to improve the outcomes of FET is of primary importance. Acupuncture is a vital portion of Traditional Chinese Medicine and is increasingly used as an adjuvant treatment for FET (7). Accumulating studies suggest that acupuncture can regulate the neuroendocrine immune system (8), improve endometrial receptivity, and reduce adverse reactions during FET (9, 10), thereby achieving better therapeutic effects. However, other randomized controlled trials (RCTs) hold distinct points, which find no significant benefits of acupuncture adjuvant to embryo transfer and the mechanism of its effects cannot be converged (11, 12).

To date, the effects and safety of acupuncture on pregnancy outcomes of FET are under controversy in clinical practice (13); however, relevant studies have placed more emphasis on fresh embryo transfer rather than FET (14, 15). Moreover, the systematic review and meta-analysis on acupuncture auxiliary to FET have not previously been reported. Therefore, we purpose to evaluate the effects and safety of acupuncture as an adjuvant treatment for infertile women undergoing FET, thus providing more evidence for further clinical practice and even experimental design.

## Methods

This systematic review and meta-analysis followed the Preferred Reporting Items for Systematic Reviews and meta-analysis (PRISMA) statement (16) (Supplementary Presentation 1) and was registered in the International Platform of Registered Systematic Review and Meta-analysis Protocols (registration number: INPLASY2021110077; Supplementary Presentation 2).

### Identification of studies

We searched for RCTs in electronic databases, such as Cochrane Library, PubMed, Embase, and the Chinese Biomedical database (SinoMed), Chinese National Knowledge Infrastructure (CNKI), and Chinese Technology Periodical Database (VIP), from inception to 30 June 2022. We also searched for previous systematic reviews on acupuncture for embryo transfer in order to review trials related to FET. In addition, the following databases of ongoing trials are retrieved: Clinicaltrials.gov, the World Health Organization's International Clinical Trials Registry Platform, and the Chinese Clinical Trial Register. We combined MeSH terms and free words in retrieval, and the search strategy of PubMed is shown in Supplementary Table 1, which is parallel to other databases.

### Inclusion criteria

We considered only RCTs that compared acupuncture with sham acupuncture or no adjuvant treatment for infertile women undergoing FET, with the objective to improve FET success rates. There were no restrictions on the reasons for FET and the protocols of FET. And trials that considered *in vitro* fertilization or intracytoplasmic sperm injection as fertilization protocols before FET were eligible. We included acupuncture performed at any time around FET (i.e., before, around, and after FET), and all types of acupuncture were considered (e.g., traditional acupuncture, electroacupuncture, and transcutaneous electrical acupuncture point stimulation). We also identified any unpublished trials that met our inclusion criteria. In addition, we included RCTs published in any language.

### Exclusion criteria

We excluded trials that assessed acupuncture as an adjuvant therapy to anesthesia for oocyte retrieval, because the purpose, protocol, and administration of these two sets of trials were far removed from each other. Besides, trials that offered insufficient and unreliable information on their methods and outcomes were excluded. We also excluded patients who were treated with co-intervention of acupuncture and herbal medicine, because it resulted in unequal co-intervention across treatment groups.

### Outcome measures

We pre-specified clinical pregnancy rate (CPR) as our primary outcome, because it was reported in more RCTs and would contribute to our major analyses. The definition of CPR was the presence of at least one gestational sac with a fetal heartbeat, which was confirmed by ultrasound 5 weeks after transfer (6). The secondary outcomes were optional: biochemical pregnancy rate, a positive hCG serum, or urine test 14 days after transfer; live birth rate, any neonate born alive after 28 weeks gestation; endometrial thickness; and endometrial pattern, regarding the triple-line pattern (Pattern A and B) as the most suitable for fertility (17).

### Data extraction

Two authors (CZ and WX) independently extracted data, with disagreements discussed and resolved with the corresponding author (QZ). The full texts of the studies would be screened for further evaluation if the titles and abstracts were eligible. We extracted characteristics of included studies with a standardized data extraction form, such as the methods, participants, interventions, control groups, outcomes, and adverse events. We contacted the corresponding authors of those trials that afforded insufficient details on their articles to obtain further information.

### Risk of bias assessment

The Cochrane risk of bias assessment tool was used to evaluate the methodological quality of included trials. The criteria were composed of the following domains: random sequence generation (selection bias), allocation concealment (selection bias), blinding of participants and personnel (performance bias), incomplete outcome data (attrition bias), selective reporting (reporting bias), and other bias. Two authors (CZ and WX) separately assessed each item as high, low, or unclear risk of bias. Any disagreements were resolved by discussion with the corresponding author (QZ) and methodological experts. Due to the particularity of acupuncture, blinding patients and physicians was difficult and the absence of blinding them was not considered a critical source of bias.

### Data synthesis and analysis

We used Review Manager 5.4 to calculate the pooled data. The outcome measures were expressed with risk ratio (RR) when acted as dichotomous data or mean difference (MD) when performed as continuous data, and a 95% confidence interval (CI). And the statistical heterogeneity between included studies was evaluated by using both the *I*^2^ statistic and the *P*-value of the χ^2^ test. According to the Cochrane Handbook (18), the following guides are suggested for interpreting *I*^2^ values: 0–40% might not be important; 30–60% may represent moderate heterogeneity; 50–90% may represent substantial heterogeneity; and 75–100% may represent considerable heterogeneity. Whether a fixed-effects model or a random-effects model was applied depends on the comprehensive analyses of statistical, clinical, and methodological heterogeneity. For our meta-analysis, if the statistical heterogeneity was moderate or above (*I*^2^ > 30%) by using the fixed-effects model, we would investigate the sources of heterogeneity through subgroup or sensibility analyses for the primary outcome and turn to the random-effects model, because the clinical heterogeneity of acupuncture protocols and settings was expected. After that, if the heterogeneity could not be explained, this review would be reported as a description of the included studies instead of data synthesis. When at least 10 studies were included, we would construct funnel plots to assess the likelihood of publication bias.

### Subgroup and sensitivity analyses

We performed subgroup analyses that might contribute to heterogeneity and impact the effects of adjuvant acupuncture on the primary outcome. (1) Type of control: Sham acupuncture or no adjuvant treatment. (2) Acupuncture sessions: Before, around, or after FET. (3) Total times of acupuncture: Less than 5 times, 5–15 times, or more than 15 times. (4) Populations: Recurrent implantation failure, anovulation, polycystic ovary syndrome, elder women (>35 years old), and unclear. We also conducted a sensitivity analysis using the leave-one-out approach: Excluding the trials which were potential contributors to heterogeneity and instability, the primary outcome would be analyzed again.

### Quality of evidence

The quality of evidence of every outcome was evaluated by the Grading of Recommendation, Assessment, Development, and Evaluation (GRADE) approach, and was classified into four levels: very low, low, moderate, or high. We used the GRADE pro-GDT to perform it by assessing the following five items: study design, risk of bias, inconsistency, indirectness, imprecision, and other considerations.

## Results

### Study selection

**Figure 1:**
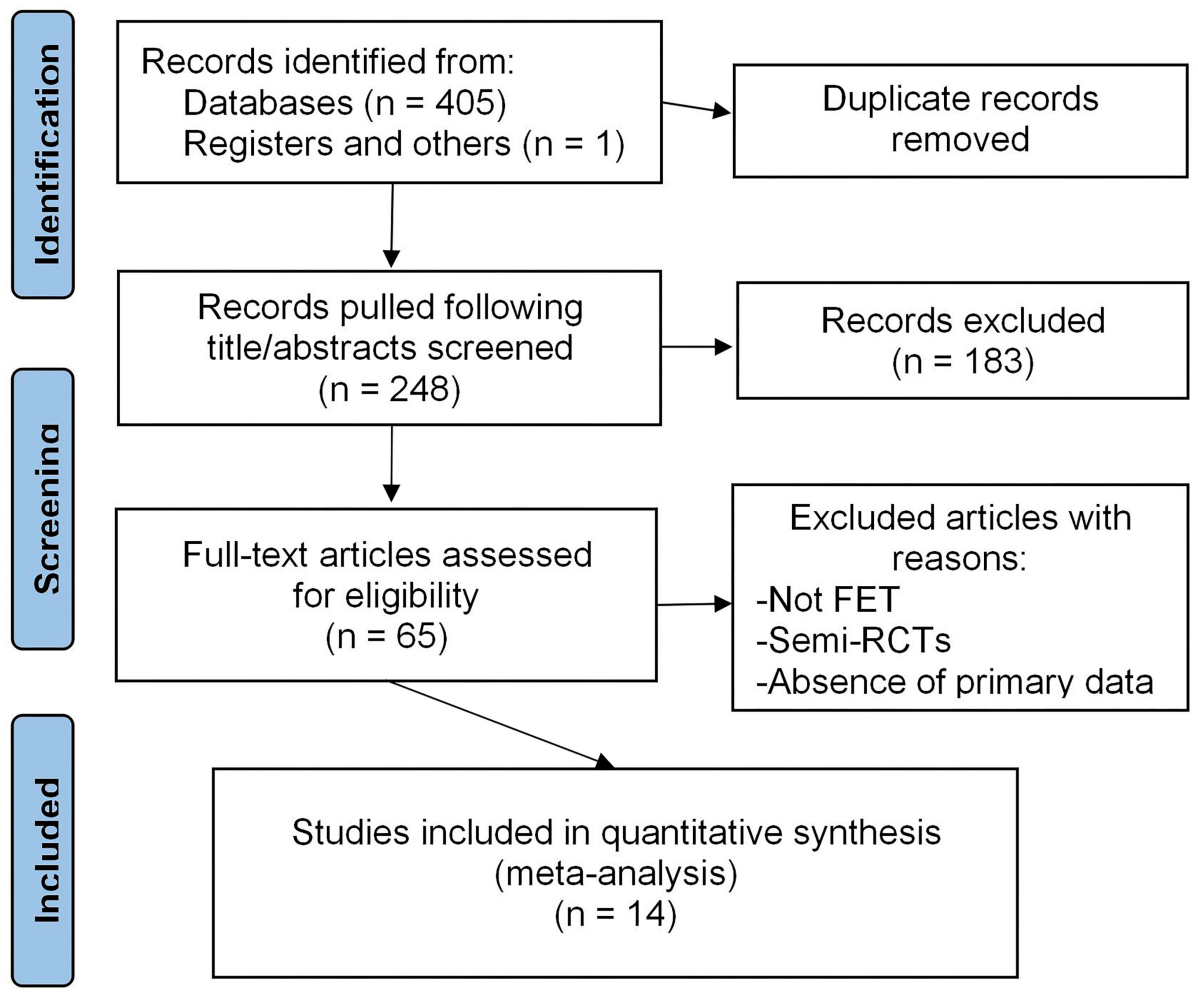
Process of study selection.

Figure 1 shows the process of study selection. With screening titles and abstracts, 183 studies were excluded because of animal experiments, reviews, comments, case reports, study protocols, non-RCTs, combination with other interventions, etc. Then, further 51 trials were excluded after full-text trials assessed for eligibility: 47 trials focused on fresh embryo transfer or mixture with fresh and frozen-thawed embryo transfer; 1 trial lacked essential data; 3 trials were semi-RCTs grouped with considering patients' selection or the order of visiting doctors. Finally, 14 RCTs with a total of 1,130 participants were included.

### Trial characteristics

The main characteristics of included trials are presented in Table 1. Despite no restrictions on the country, all 14 trials were performed in China. In all trials, women received acupuncture as an adjuvant treatment for FET. And the protocols and settings of acupuncture, which retained 25–30 min every time and lasted for 1–3 menstrual cycles, were designed for the sole objective of improving pregnancy outcomes. In terms of control types, 12 trials conducted no adjuvant treatment control (9, 10, 19–28), whereas the other 2 trials used sham acupuncture control (11, 29). Moreover, 3 trials applied electroacupuncture (19, 20, 27), 2 trials adopted warm needle moxibustion (22, 23), 3 trials employed transcutaneous electrical acupoint stimulation (21, 26, 29), 3 trials used traditional acupuncture (10, 11, 28), and the remaining 3 trials contained traditional acupuncture and warm needle moxibustion (9, 24, 25). Furthermore, 10 trials reported the DeQi sensation (9–11, 19, 20, 22, 24, 25, 27, 28), which referred to a soreness, numbness, or distension around the puncture sites or sometimes propagation along the corresponding meridians and indicated the correct needle insertion, whereas other 4 trials did not report DeQi sensation (21, 23, 26, 29), and 3 of them applied TEAS without needle insertion. Of all the trials, one was a three-arm trial (19) and the other was a four-arm trial (26), and we only extracted groups that met our inclusion criteria. For all the trials, baseline characteristics were reported as comparable between the randomized groups.

**Table 1:** Characteristics of included trials.

**References**	**N(I/C)**	**Mean age** **(I/C; y)**	**Duration of infertility** **(I/C; y)**	**Acupuncture**	**Control**	**Sessions; Frequencies; Cycles^a^**	**FET outcomes**
Chen et al. (20)	94 (44/50)	32.07/33.59	6.26/7.08	EA	No adjuvant treatment	Around FET; qd, starting on day 5, and only 1 time after FET; 1	CPR, ET
Deng et al. (27)	100 (49/51)	35.52/35.68	Unclear	EA	No adjuvant treatment	Before FET; qod, after menstruation and until FET; 1	CPR, ET
Feng (21)	79 (39/40)	31.21/30.10	3.18/3.90	TEAS	No adjuvant treatment	Around FET; 24 h before FET and 0.5–4 h after FET; 1	CPR, BPR, ET, EP
Liang et al. (26)	83 (43/40)	31.86/31.98	Unclear	TEAS	No adjuvant treatment	Around FET; qd, 3 days; 1	CPR, BPR, ET
Liu and Tang (23)	63 (31/32)	38.42/39.38	7.57/7.05	WNM	No adjuvant treatment	Before FET; qd, starting on day 10; 3	CPR, BPR, ET, EP
Ma et al. (22)	60 (30/30)	30.00/31.00	4.10/4.70	WNM	No adjuvant treatment	Around FET; qd, starting on day 2; 1	CPR, ET, EP
Shuai et al. (29)	68 (34/34)	29.47/29.65	4.56/3.88	TEAS	Sham^b^ TEAS	Before FET; qod, starting on day 3, 6 times per cycle; 3	CPR, LBR, ET, EP
So et al. (11)	226 (113/113)	36.00/36.00	5.00/5.00	TA	Sham TA	After FET; immediately after FET; 1	CPR, LBR
Xing et al. (28)	68 (34/34)	34.85/34.24	4.64/4.94	TA	No adjuvant treatment	Around FET; tiw, performing FET in the third cycle; 3	CPR, LBR
Xu et al. (10)	60 (30/30)	31.00/30.00	3.30/2.96	TA	No adjuvant treatment	Before FET; qod, starting on day 2; 1	CPR, ET, EP
Xue et al. (9)	74 (37/37)	35.00/34.00	5.00/5.20	TA/WNM	No adjuvant treatment	Around FET; qod, starting on day 5, until 14 days after FET or next menstruation; 1–3	CPR, ET, EP
Yang (24)	60 (30/30)	32.07/33.00	5.97/5.20	TA/WNM	No adjuvant treatment	Before FET; tiw, until FET; 2	CPR, BPR, ET
Zhang et al. (19)	26 (14/12)	30.30/32.50	5.80/4.50	EA	No adjuvant treatment	Before FET; qod, after menstruation and until FET; 1	CPR, ET
Zhuang (25)	69 (34/35)	34.29/34.26	4.38/4.20	TA/WNM	No adjuvant treatment	Around FET; tiw, performing FET in the third cycle; 3	CPR, BPR, LBR, ET, EP

N(I/C), Number of participants (Intervention / Control); Y, Year; qd, once a day; qod, once every other day; tiw, 3 times a week; EA, electroacupuncture; TA, traditional acupuncture; TEAS, transcutaneous electrical acupuncture point stimulation; WNM, warm needle moxibustion; CPR, clinical pregnancy rate; BRP, biochemical pregnancy rate; LBR, live birth rate; ET, endometrial thickness; EP, endometrial pattern.

a The menstrual cycles that acupuncture treatment lasted for, regardless of acupuncture frequencies and times in each cycle.

b For all sham-controlled trials, the same acupoints were applied as in the true acupuncture group.

### Methodological quality of included trials

**Figure 2:**
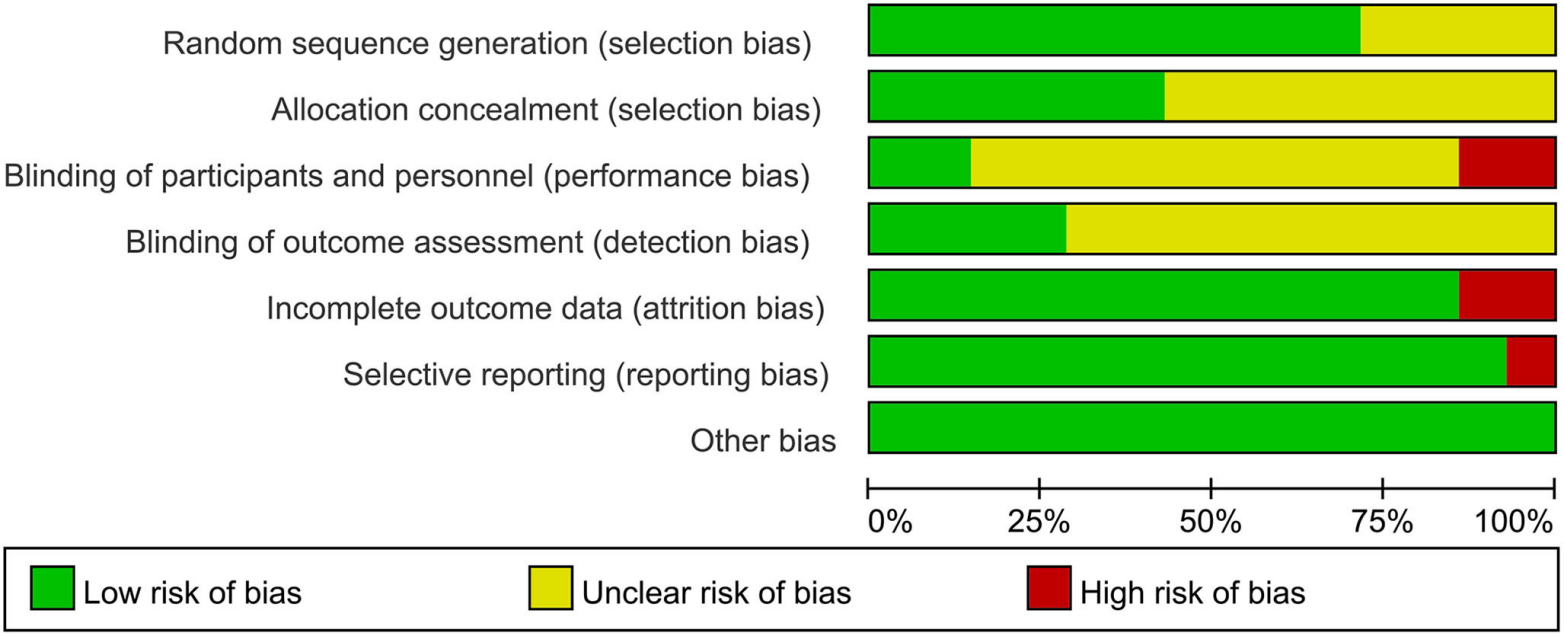
Risk of bias assessment.

Figure 2 demonstrates the assessment for the risk of bias. For 10 trials (9–11, 19, 22, 24–26, 28, 29), adequate information about random sequence generation was reported. For the other 4 trials (20, 21, 23, 27), the details of the random sequence were not available, and we assessed them as unclear risk of selection bias; however, the randomization seemed to be successful because of no baseline differences between groups. A total of 8 of all trials did not provide adequate allocation concealment, but there was also baseline resemblance between the compared groups (19–23 and 26–28). Due to the particularity of acupuncture, blinding patients and physicians was difficult, therefore only two trials (11, 29) performed blinding of patients resulting in a low risk of performance bias, another two trials (24, 25) clearly reported no blinding, and the rest 10 trials (9, 10, 19–23, 26–28) did not mention whether blinding was adopted or not. In 4 trials (9, 11, 24, 25), blinding of outcome assessment was applied, whereas the other 10 trials (10, 19–23, 26–29) had no sufficient information about it. For incomplete outcome data, 12 trials (9–11, 20–25, 27–29) had no attrition bias for missing patients or data; however, 2 trials (19, 26) did not report why it appeared the unequal numbers of patients when randomly grouped (intervention/control = 14/12 and 43/40, respectively).

### Efficacy analyses

**Figure 3:**
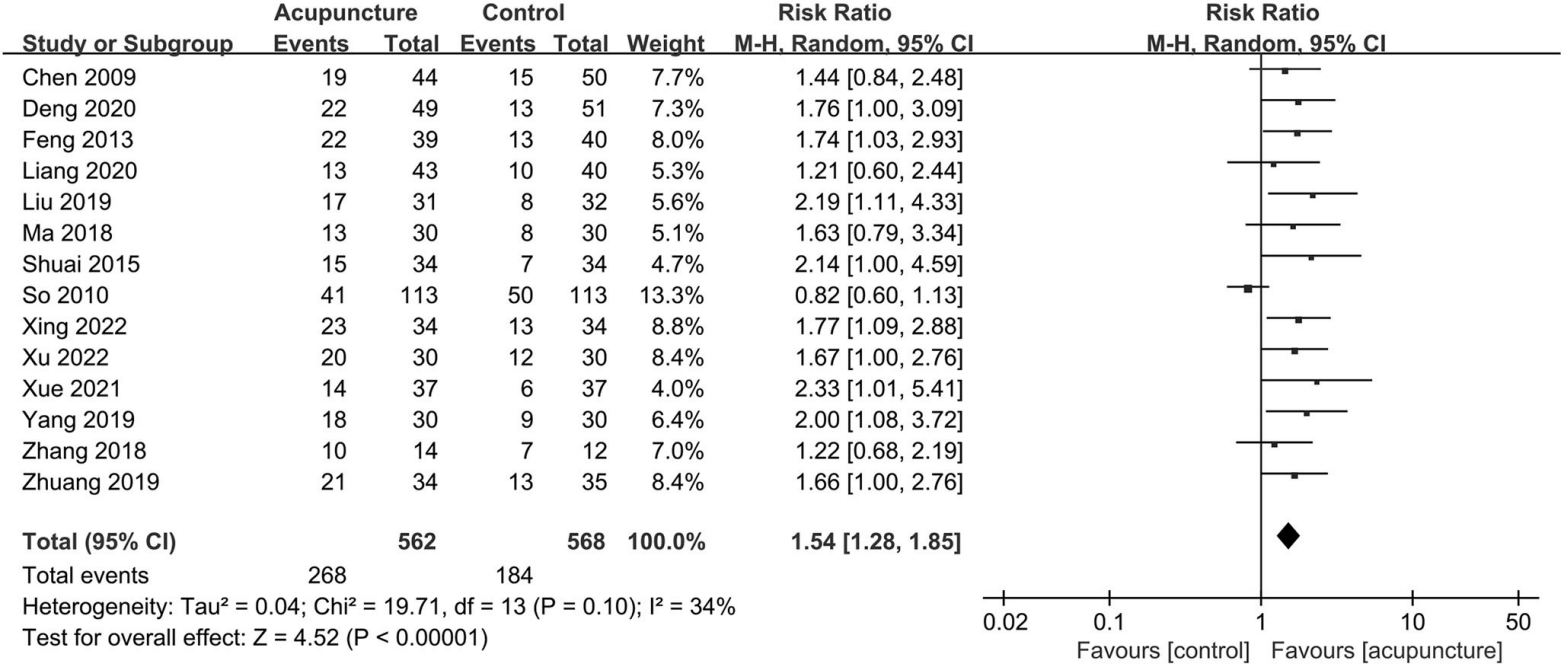
Forest plot of clinical pregnancy rate.

**Figure 4:**
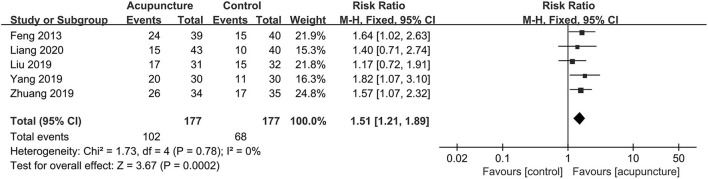
Forest plot of biochemical pregnancy rate.

**Figure 5:**
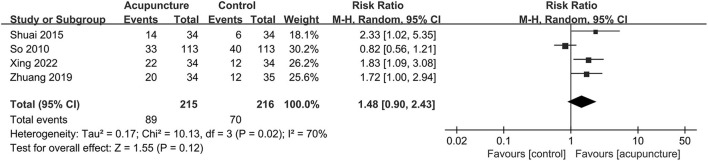
Forest plot of live birth rate.

The clinical pregnancy rate, which was considered the primary outcome in our study and was reported in all included trials, had significant pooled benefits of acupuncture adjuvant to FET with statistically moderate heterogeneity (RR = 1.54, 95% CI [1.28, 1.85], *P* < 0.00001; *I*^2^ = 34%; Figure 3). For biochemical pregnancy rate involved in five trials (21, 23–26), we also found significant effects of acupuncture and the heterogeneity might not be important (RR = 1.51, 95% CI [1.21, 1.89], *P* = 0.0002; *I*^2^ = 0; Figure 4). However, for live birth rate, which were reported in four trials (11, 25, 28, 29), there was no statistical difference between the two groups, and the heterogeneity was substantial (RR = 1.48, 95% CI [0.90, 2.43], *P* = 0.012; *I*^2^ = 72; Figure 5).

**Figure 6:**
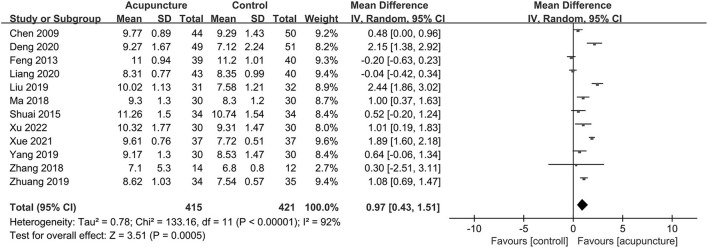
Forest plot of endometrial thickness.

**Figure 7:**
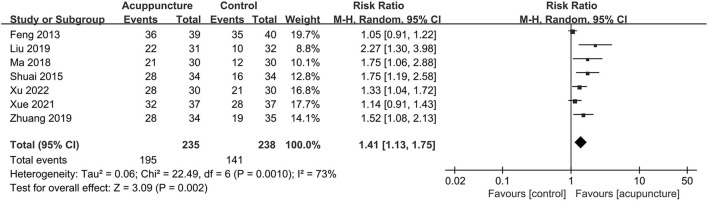
Forest plot of endometrial pattern.

In addition, the endometrial thickness reported in 12 trials (9, 10, 19–27, 29) was statistically increased, and the heterogeneity was substantial (MD = 0.97, 95% CI [0.43, 1.51], *P* = 0.0005; *I*^2^ = 92%; Figure 6). Besides, seven trials (9, 10, 21–23, 25, 29) measured endometrial pattern, and we found the acupuncture could significantly promote the number of trilinear endometrium (Patterns A and B) with substantial heterogeneity (RR = 1.41, 95% CI [1.13, 1.75], *P* = 0.002; *I*^2^ = 73%; Figure 7).

### Sources of heterogeneity

To investigate the sources of heterogeneity, we combined all 14 trials for subgroup analyses on the primary outcome (clinical pregnancy rate), and the variables that might impact the effects of adjuvant acupuncture on FET were evaluated. The analyses showed that sham-controlled trials and trials of performing acupuncture <5 times had substantial heterogeneity (*I*^2^ = 81 and 66%, respectively). Moreover, the above two variables, the trials of conducting acupuncture only after FET, and the trials of not mentioning a specific population (unclear) indicated no significant effects (*P* = 0.65, 0.22, 0.57, and 0.08; respectively; Table 2; Supplementary Figures 1–4).

**Table 2:** Subgroup analyses on clinical pregnancy rate (random-effects model, *n* = 14).

**Subgroups**	**Risk ratio [95% CI]**	**Coefficient**	**Heterogeneity**	
		***P*-value**	***I^2^* (%)**	***P*-value**
**(1) Type of control**				
Sham acupuncture (*n* = 2)	1.24 [0.49, 3.18]	0.65	81	0.02
No adjuvant treatment (*n* = 12)	1.67 [1.41, 1.97]	<0.00001	0	0.97
**(2) Acupuncture sessions**				
Before FFT (*n* = 6)	1.75 [1.37, 2.23]	<0.00001	0	0.78
Around FET (*n* = 7)	1.64 [1.32, 2.05]	<0.0001	0	0.94
After FET (*n* = 1)	0.82 [0.60, 1.13]	0.22		
**(3) Total times of acupuncture**				
Less than 5 times (*n* = 3)	1.15 [0.70, 1.90]	0.57	66	0.05
5-15 times (*n* = 5)	1.59 [1.24, 2.04]	0.0003	0	0.81
More than 15 times (*n* = 6)	1.86 [1.44, 2.40]	<0.00001	0	0.96
**(4) Populations**				
Recurrent implantation failure (*n* = 6)	1.57 [1.57, 1.21]	0.0006	0	0.8
Anovulation (*n* = 1)	1.67 [1.00, 2.76]	0.05		
Polycystic ovary syndrome (*n* = 1)	1.77 [1.09, 2.88]	0.02		
Elderly women (> 35 years old) (*n* = 1)	2.19 [1.11, 4.33]	0.02		
Unclear (*n* = 5)	1.46 [0.96, 2.22]	0.08	68	0.1

We also carried out a sensitivity analysis to further interpret the heterogeneity and detect instability. When we excluded the So (2010) trial (11), the statistical heterogeneity decreased to be not important (*I*^2^ = 0%), and the pooled benefits were stable (RR = 1.69, 95% CI [1.43, 1.99]; Supplementary Figure 5).

### Publication bias

**Figure 8:**
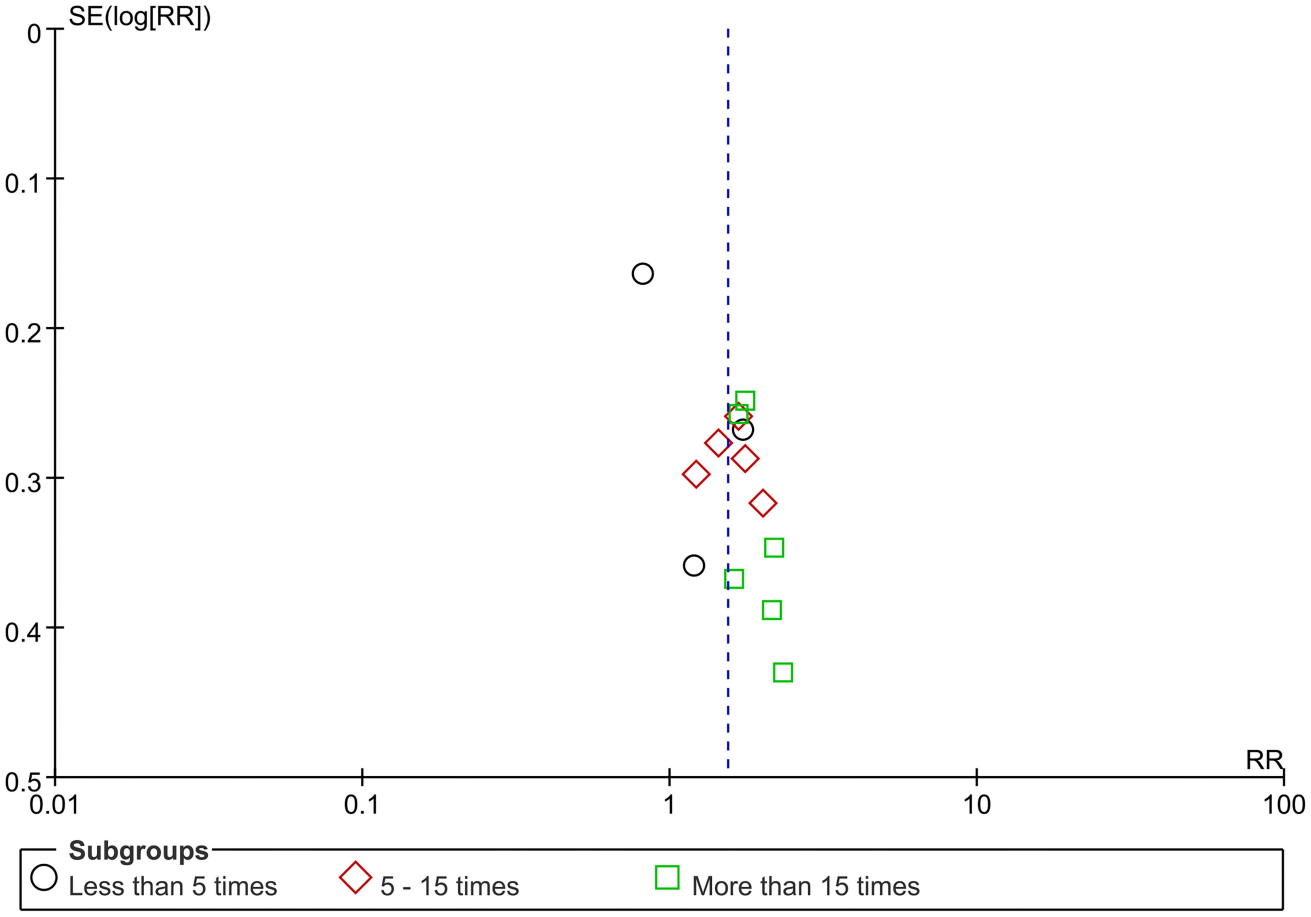
Funnel plot of clinical pregnancy rate.

The funnel plot (Figure 8) including all 14 trials on clinical pregnancy rate was asymmetric.

### Adverse events

A total of four trials (11, 19, 24, 25) reported adverse events, such as nausea or dizziness, but none of these trials mentioned any serious adverse events associated with acupuncture and hindered subsequent treatment.

### Certainty of evidence

By using the GRADE pro-GDT, the evidence of clinical pregnancy rate was low certainty, indicating our confidence in the effect estimate was limited. For other outcomes, the level of evidence ranged from low to moderate, and more details were available in Table 3.

**Table 3:** Certainty of evidence (GRADE).

**Outcomes**	**Anticipated absolute effects** ^*^ **(95% CI)**		**Relative effect (95% CI)**	**No. of participants (trials)**	**Certainty of the evidence (GRADE)**
	**Risk with control**	**Risk with acupuncture**			
Clinical pregnancy rate	324 per 1,000	499 per 1,000 (415–599)	RR 1.54 (1.28–1.85)	1130 (14 RCTs)	⊕⊕○○ Low^a^, ^b^
Biochemical pregnancy rate	384 per 1,000	580 per 1,000 (465–726)	RR 1.51 (1.21–1.89)	354 (5 RCTs)	⊕⊕○○ Low^a^, ^c^
Live birth rate	324 per 1,000	480 per 1,000 (292–788)	RR 1.48 (0.90–2.43)	431 (4 RCTs)	⊕⊕○○ Low^a^, ^d^
Endometrial thickness	–	MD 0.97 higher (0.43 higher−1.51 higher)	–	836 (12 RCTs)	⊕⊕⊕○ Moderate^a^
Endometrial pattern	592 per 1,000	835 per 1,000 (669–1,000)	RR 1.41 (1.13–1.75)	413 (7 RCTs)	⊕⊕⊕○ Moderate^a^

* The risk in the intervention group (and its 95% confidence interval) is based on the assumed risk in the comparison group and the relative effect of the intervention (and its 95% CI).

a Part of the trials did not provide details on allocation concealment or blinding;

b Only a few negative results were reported;

c The sample size was small;

d Only a few trials reported the outcome and the 95%CI was wide.

## Discussion

The past few years have seen acupuncture as an adjuvant therapy to FET for infertile women become a hot-button issue that has sparked numerous studies in the reproductive field. However, the effects and safety of acupuncture on the outcomes of FET are highly controversial (12, 13).

### Main findings

Obtaining pregnancy is the ultimate objective of acupuncture auxiliary to FET. Moreover, a moderate endometrial thickness (between 7 and 14 mm) with the triple-line pattern (Pattern A and B) also contributes directly to embryo implantation, thus improving pregnancy outcomes (17).

In the study, we found significant pooled effects of acupuncture adjuvant to FET on the clinical pregnancy rate and biochemical pregnancy rate, suggesting that acupuncture treatment could improve the FET success rates. Acupuncture might act on the hypothalamic–pituitary–ovarian axis and target the uterus (30), up-regulate the IRS-1/PI3K/GLUT4 signaling pathway to promote embryonic development potential in patients with PCOS-IR (31), alleviate Shen deficiency syndrome, and increase the clinical pregnancy during embryo transfer (32). Besides, it was observed that three sessions of acupuncture around embryo transfer significantly improved pregnancy outcomes and reduced anxiety levels in women with unexplained infertility (33).

Furthermore, our study showed that acupuncture was also significantly beneficial in the endometrial thickness and triple-line pattern during FET, indicating that this therapy could improve endometrium receptivity, thus increasing pregnancy rates. It was predicted that hsa-miR-449a, hsa-miR-3135b, and hsa-miR-345-3p might be associated with the mechanisms that acupuncture contributed to endometrium receptivity to prepare for embryo transfer (34); and reviews also reported that acupuncture had effects on endometrial receptivity and pregnancy outcomes of recurrent implantation failure (35, 36).

In addition, although acupuncture had no statistical effects on the live birth rate of FET, it also showed an increasing trend. This might be tied up with the fact that only four included trials reported the outcome, and the sample size was too small to obtain accurate effects.

### Heterogeneity analyses

Most of our outcomes were accompanied by moderate or substantial heterogeneity. Therefore, we performed subgroup analyses to explore the sources of heterogeneity, combining all 14 trials on the primary outcome. The analyses showed most variables served as significant catalysts for the effectiveness of acupuncture except for four variables, which were sham-controlled and performed acupuncture only after FET or <5 times, and had an unclear population.

According to related studies (37–39), the sham-controlled trials had a risk that sham acupuncture might have acupuncture-specific effects on pregnancy outcomes *via* the identical mechanism of true acupuncture, especially when sham needles were placed at the true acupuncture points, suggesting that the sham acupuncture was not an inert control and the effects of true acupuncture might be underestimated, as was the case in the two sham-controlled trials (11, 29) included in our study. Besides, as was mentioned before, acupuncture could improve endometrial morphology and thereby increase pregnancy rates, while the subgroup that performed needles only after FET might have fewer efficacies on pregnancy outcomes than other variables, as was the case in the trial (11) included in this review. Moreover, there was no significant effect in the subgroup of conducting acupuncture <5 times, which might imply that fewer acupuncture times had insufficient efficacy on pregnancy outcomes of FET. Finally, it showed no statistical effect in the subgroup of the unclear population, which might result from therapeutic protocols and doctor–patient interactions, and so on (40). Nevertheless, we found acupuncture adjuvant to FET could be significantly beneficial to the populations such as recurrent implantation failure, polycystic ovary syndrome, and elderly women.

These above-mentioned four variables were involved in the same trial (11) and might be the source of heterogeneity. So, we removed it for a sensitivity analysis and then found the statistical heterogeneity reduced to be not important and the pooled benefit of acupuncture on the primary outcome was stable, which might further interpret most of the heterogeneity in the results.

### Publication bias

The funnel plot on clinical pregnancy rate showed asymmetrically, which might indicate publication bias. Generally, the asymmetric resulted from more effects in smaller trials, and positive results were easier to be published. Besides, language and regional bias could also invite it; of all included trials, two were published in English (11, 29) and others in Chinese (9, 10, 19–28), and all trials were conducted in China. Moreover, clinical heterogeneity such as different acupuncture protocols between trials was also a potential contributor to asymmetry. Finally, although we attempted to gain any unpublished or unidentified trials with negative results, none were located, which was possibly responsible for the funnel plot asymmetry as well (38).

### Safety of acupuncture

Acupuncture is a mild treatment with relatively fewer adverse reactions and costs. Of all included trials, only four reported adverse events such as nausea or dizziness, and it could be concluded that adverse events associated with acupuncture were extremely limited and no serious reactions were reported. Although acupuncture has very few risks of severe adverse events, physicians can prevent most adverse reactions through specialized knowledge and administration. And the expert consensus (41) also suggested that electroacupuncture or transcutaneous electrical acupuncture point stimulation (TEAS) combined with assisted reproductive technique might be regarded as a model of the integrated application of traditional Chinese medicine and modern medicine; in particular, TEAS is widely favored by patients owing to its advantages of easy-operation, non-invasive nature, and painless procedures.

### Quality of evidence

The quality of evidence of all outcomes ranged from low to moderate. According to the GRADE Working Group (https://www.gradepro.org/), low certainty indicates the true effect may be substantially different from the estimate of the effect; and moderate certainty suggests the true effect is likely to be close to the estimate of the effect, but there is a possibility that it is substantially different. In our study, the reasons for the downgrades of evidence level mainly contained the following two: Part of the trials did not provide details on allocation concealment or blinding, and only a few negative results were reported. In fact, due to the particularity of acupuncture, blinding patients and physicians was difficult, which should not be considered as a critical source of bias (24). Although the results showed significant pooled benefits of acupuncture in comparison to the control group, eligible clinical trials with high methodological quality are needed to improve the level of evidence.

### Limitations

The following two are taken as the major limitations in our study: The quality of included trials and reporting bias. First, part of included trials have insufficient information about random sequence generation, allocation concealment, or blinding of outcome assessment, and have shown small sample sizes, which result in low methodological quality and, therefore, downgrade the evidence level. Furthermore, some types of reporting bias cannot be excluded in this review, such as publication bias, language, and regional bias, which can potentially invite limitations, and thereby it is difficult to obtain accurate results.

## Conclusion

Acupuncture could be instrumental in the pregnancy outcomes of FET, and the quality of evidence ranges from low to moderate. Nowadays, acupuncture has been undertaken widely in China and other nations, and has very few risks of severe adverse events. However, trials with small sample sizes and low methodological quality lead to uncertain findings. Therefore, for future studies, RCTs with rigorous methodological quality on a large scale should be considered, and the protocols of acupuncture also need more investigations (e.g., appropriate control groups, sessions, and times). In addition, when performing acupuncture as an adjuvant treatment for *in vitro* fertilization– embryo transfer, the differences between FET and fresh cycles deserve further research.

## Data availability statement

The original contributions presented in the study are included in the article/Supplementary material, further inquiries can be directed to the corresponding author.

## Author contributions

CZ and WX designed the study, conducted this analysis, and wrote and edited the manuscript. JH and XZ participated in selecting articles and performing the quality assessment. FL, XY, and JM helped to search the literature and analyzed the data. QZ proposed the study, explained the data, and revised the report. All authors provided comments on the manuscript and approved its final version.

## Funding

This study was supported by the Sichuan Province's Science and Technology Project Fund of China (No. 2020YFSY0043) and the National Natural Science Foundation of China (Nos. 82174430 and 81973901).

## Conflict of interest

The authors declare that the research was conducted in the absence of any commercial or financial relationships that could be construed as a potential conflict of interest.

## Publisher's note

All claims expressed in this article are solely those of the authors and do not necessarily represent those of their affiliated organizations, or those of the publisher, the editors and the reviewers. Any product that may be evaluated in this article, or claim that may be made by its manufacturer, is not guaranteed or endorsed by the publisher.
