# Supplementary material for: Effects of acupuncture on the pregnancy outcomes of frozen-thawed embryo transfer: A systematic review and meta-analysis
Source: Front Public Health. 2022 Sep 9;10:987276. doi: 10.3389/fpubh.2022.987276 (PMC9501879; doi:10.3389/fpubh.2022.987276)
Supplement: Supplementary file 1 [file Table_1.DOCX]

## Supplementary Table 1

| **Database** | **Retrieval strategy** |
| --- | --- |
| PubMed | #1 ((((((((((Acupuncture Therapy[MeSH Terms]) OR (Acupuncture Treatment)) OR (Acupuncture Treatments)) OR (Treatment, Acupuncture)) OR (Therapy, Acupuncture)) OR (Pharmacoacupuncture Treatment)) OR (Treatment, Pharmacoacupuncture)) OR (Pharmacoacupuncture Therapy)) OR (Therapy, Pharmacoacupuncture)) OR (Acupotomy)) OR (Acupotomies) |
|  | #2 ((((((((((((Embryo Transfer[MeSH Terms]) OR (Embryo Transfers)) OR (Transfer, Embryo)) OR (Transfers, Embryo)) OR (Blastocyst Transfer)) OR (Tubal Embryo Transfer)) OR (Tubal Embryo Stage Transfer)) OR (Single Embryo Transfer)) OR (Frozen embryo transfer)) OR (Frozen-thawed embryo)) OR (Frozen-thawed embryo transfer)) OR (Transfer, Frozen-thawed embryo)) OR (Transfer, Frozen embryo) |
|  | #3 #1 AND #2 |

## Retrieval strategy of PubMed and parallel to other databases
