# Supplementary material for: Effects of acupuncture on the pregnancy outcomes of frozen-thawed embryo transfer: A systematic review and meta-analysis
Source: Front Public Health. 2022 Sep 9;10:987276. doi: 10.3389/fpubh.2022.987276 (PMC9501879; doi:10.3389/fpubh.2022.987276)
Supplement: Supplementary file 2 [file Presentation_1.PDF]

PRISMA 2020 Checklist

Section and Topic	Item #	Checklist item	Location where item is reported
**TITLE**			
Title	1	Effects of Acupuncture on the Pregnancy Outcomes of Frozen-thawed Embryo Transfer: A Systematic Review and Meta-analysis	Page 1
**ABSTRACT**			
Abstract	2	**Background, Methods, Results, Conclusion** and **Keywords**	Page 1-2, line 13-40
**INTRODUCTION**			
Rationale	3	Described in the **1 Introduction**	Page 2, line 41-57
Objectives	4	Provided in the **1 Introduction**	Page 2, line 58-63
**METHODS**			
Eligibility criteria	5	Presented in the **2.2 Inclusion Criteria**	Page 3, line 79-88
Information sources	6	Presented in the **2.1 Identification of studies**	Page 2-3, line 69-76
Search strategy	7	Presented in the **2.1 Identification of studies**	Page 2-3, line 76-78
Selection process	8	Presented in the **2.2 Inclusion Criteria; 2.3 Exclusion Criteria**	Page 3, line 79-95
Data collection process	9	Presented in the **2.5 Data extraction**	Page 3, line 104-110
Data items	10a	Presented in the **2.4 Outcome Measures**	Page 3, line 96-103
	10b	Presented in the **2.4 Outcome Measures**	Page 3, line 96-103
Study risk of bias assessment	11	Provided in the **2.6 Risk of bias assessment**	Page 3-4, line 111-119
Effect measures	12	Provided in the **2.7 Data synthesis and analysis**	Page 4, line 120-124
Synthesis methods	13a	Described in the **2.7 Data synthesis and analysis**	Page 4, line 125-136
	13b	Described in the **2.7 Data synthesis and analysis**	Page 4, line 125-136
	13c	Described in the **2.7 Data synthesis and analysis**	Page 4, line 125-136
	13d	Described in the **2.7 Data synthesis and analysis**	Page 4, line 125-136
	13e	Described in the **2.8 Subgroup and sensitivity analyses**	Page 4, line 137-143
	13f	Described in the **2.8 Subgroup and sensitivity analyses**	Page 4, line 137-143
Reporting bias assessment	14	Described in the **2.6 Risk of bias assessment**	Page 3-4, line 111-119
Certainty assessment	15	Described in the **2.9 Quality of evidence**	Page 4, line 144-149
**RESULTS**			
Study selection	16a	Described in the **3.1 Study selection**	Page 4-5, line 151-158
	16b	Described in the **3.1 Study selection**	Page 4-5, line 151-158
Study characteristics	17	Described in the **3.2 Trial characteristics**	Page 5, line 159-175
Risk of bias in	18	Described in the **3.3 Methodological quality of included trials**	Page 5, line 176-190

PRISMA 2020 Checklist

Section and Topic	Item #	Checklist item	Location where item is reported
studies			
Results of individual studies	19	Involved in the **Figures 3-7**	
Results of syntheses	20a	Described in the **3.4 Efficacy analyses**	Page 5-6, line 191-204
	20b	Described in the **3.4 Efficacy analyses**	Page 5-6, line 191-204
	20c	Presented in the **3.5 Sources of heterogeneity**	Page 6, line 205-212
	20d	Presented in the **3.5 Sources of heterogeneity**	Page 6, line 213-216
Reporting biases	21	Described in the **3.6 Publication bias**	Page 6, line 217-218
Certainty of evidence	22	Presented in the **3.8 Certainty of evidence**	Page 6, line 223-226
**DISCUSSION**			
Discussion	23a	Provided in the **4.1 Main findings**; **4.2 Heterogeneity analyses; 4.3 Publication Bias; 4.4 Safety of acupuncture**	Page 6-8, line 227-293
	23b	Discussed in the **4.5 Quality of evidence** and **4.6 Limitation**	Page 8, line 294-313
	23c	Discussed in the **4.6 Limitation**	Page 8, line 306-313
	23d	Presented in the **5 Conclusion**	Page 8-9, line 314-320
**OTHER INFORMATION**			
Registration and protocol	24a	Provided in the **Supplementary File 2 (Number: INPLASY:2021110077)**	Supplementary material
	24b	Provided in the **Supplementary File 2**	
	24c	The protocol was updated two times for adding two additional outcomes and extending retrieval deadlines.	--
Support	25	Sichuan Province's Science and Technology Project Fund of China (No.2020YFSY0043).	Page 9, line 329-331
Competing interests	26	None declared	Page 9, line 332-333
Availability of data, code and other materials	27	Included in the **Data availability Statement**	Page 9, line 321-323

From: Page MJ, McKenzie JE, Bossuyt PM, Boutron I, Hoffmann TC, Mulrow CD, et al. The PRISMA 2020 statement: an updated guideline for reporting systematic reviews. BMJ 2021;372:n71. doi: 10.1136/bmj.n71

For more information, visit: http://www.prisma-statement.org/
