# Supplementary material for: Effects of acupuncture on the pregnancy outcomes of frozen-thawed embryo transfer: A systematic review and meta-analysis
Source: Front Public Health. 2022 Sep 9;10:987276. doi: 10.3389/fpubh.2022.987276 (PMC9501879; doi:10.3389/fpubh.2022.987276)
Supplement: Supplementary file 3 [file Presentation_2.PDF]

INPLASY PROTOCOL

To cite: Zhu et al. Effects of Acupuncture on the Pregnancy Outcomes of Frozen-thawed Embryo Transfer: A Protocol of Systematic Review and Meta-analysis. Inplasy protocol 2021110077. doi: 10.37766/inplasy2021.11.0077

Received: 21 November 2021

Published: 21 November 2021

Corresponding author:
Can Zhu

zhucan@stu.cdutcm.edu.cn

Author Affiliation:
Hospital of Chengdu University of Traditional Chinese Medicine; Guizhou University of Traditional Chinese Medicine.

Support: **2020YFSY0043.**

Review Stage at time of this submission: **The review has not yet started.**

Conflicts of interest:
None declared.

Effects of Acupuncture on the Pregnancy Outcomes of Frozen-thawed Embryo Transfer: A Protocol of Systematic Review and Meta-analysis

Zhu, C[1]; Xia, WT[2]; Huang, JZ[3]; Zhang, X[4]; Li, FY[5]; Yu, XR[6]; Ma, JM[7]; Zeng, Q[8].

Review question / Objective: **To determine the effects of acupuncture as an adjuvant to frozen-thawed embryo transfer(FET) for infertile women.**
Condition being studied: **FET is applied among infertile women who suffer from implantation failure or cycle cancellation in previous embryo transfer cycle and have at least one eligible frozen embryo. Acupuncture is commonly undertaken during FET although its role in improving pregnancy outcomes is still controversial. However, there is hardly any systematic review on acupuncture for FET to obtain a confirmative conclusion. Therefore, it is necessary to perform the meta-analysis.**

INPLASY registration number: This protocol was registered with the International Platform of Registered Systematic Review and Meta-Analysis Protocols (INPLASY) on 21 November 2021 and was last updated on 03 July 2022 (registration number INPLASY2021110077).

INTRODUCTION

Review question / Objective: **To determine the effects of acupuncture as an adjuvant to frozen-thawed embryo transfer(FET) for infertile women.**

Condition being studied: **FET is applied among infertile women who suffer from implantation failure or cycle cancellation in previous embryo transfer cycle and have at least one eligible frozen embryo. Acupuncture is commonly undertaken during FET although its role in improving**

Zhu et al. Inplasy protocol 2021110077. doi:10.37766/inplasy2021.11.0077 Downloaded from https://inplasy.com/inplasy-2021-11-0077/

pregnancy outcomes is still controversial. However, there is hardly any systematic review on acupuncture for FET to obtain a confirmative conclusion. Therefore, it is necessary to perform the meta-analysis.

METHODS

Participant or population: We include infertile women undergoing FET, without restrictions on the reasons for FET. Participants with any medical illness deemed a contraindication for FET or acupuncture treatment will be excluded.

Intervention: The intervention is acupuncture combined with FET, including any type of acupuncture at any or all time points before, during, or after FET with the intention to improve the FET outcome.

Comparator: Sham acupuncture or no adjuvant treatment during FET.

Study designs to be included: Only randomized controlled trials(RCTs) will be included. Quasi-randomised controlled trials or crossover randomised controlled trials that do not provide pre-crossover data will be excluded.

Eligibility criteria: We will only include RCTs that compare acupuncture with sham acupuncture or no adjuvant treatment during FET. And the source of stimulation include not only manual acupuncture but also electroacupuncture, moxibustion with warming needle, transcutaneous electrical acupoint stimulation(TEAS), etc. Eligible papers require the main outcome recommended as clinical pregnancy rate.

Information sources: We search for RCTs in the following electronic databases, including Cochrane Library, PubMed, Embase, and the Chinese Biomedical database (SinoMed), Chinese National Knowledge Infrastructure (CNKI), and Chinese Technology Periodical Database (VIP), from inception to June 30, 2022. We also search for previous systematic reviews on acupuncture for assisted reproductive technology in order to review trials related to FET. Additionally, the following databases of ongoing trials are retrieved: Clinicaltrials.gov, the World Health Organization's International Clinical Trials Registry Platform, and Chinese Clinical Trial Register.

Main outcome(s): Clinical pregnancy rate(CPR), defined as the presence of at least one gestational sac with fetal heartbeat, and confirmed by ultrasound 4-6 weeks after FET.

Additional outcome(s): Biochemical pregnancy rate (BPR), a positive hCG serum or urine test 14 days after transfer; live birth rate (LBR), any neonate born alive after 28 weeks gestation; endometrial thickness (ET); endometrial pattern (EP), regarding the triple-line pattern (Pattern A and B) as the most suitable for fertility.

Quality assessment / Risk of bias analysis: The Cochrane risk of bias assessment tool is used to assess the following factors: random method, allocation concealment, blinding, completeness of the data, selective reporting and other bias. Two researchers conduct independent evaluations, with any disagreements discussed and resolved with the third researcher. Due to the particularity of acupuncture that blinding of patients and physicians is difficult, absence of blinding to them is not considered a critical source of bias.

Strategy of data synthesis: The data will be pooled for meta-analysis with RevMan5.4 software. The outcome measures are expressed with risk ratio (RR) when acted as dichotomous data, or mean difference (MD) when performed as continuous data, and a 95% confidence interval (CI). And the statistical heterogeneity between included studies is evaluated by using both the I2 statistic and the P-value of the x2 test. The following guide interpreting I2 values is suggested by Cochrane Handbook: 0-40% might not be important; 30-60% may represent moderate heterogeneity; 50-90% may represent substantial heterogeneity; and 75-100% may represent considerable heterogeneity. Whether a fixed-effects model or a random-effects model is applied

Zhu et al. Inplasy protocol 2021110077. doi:10.37766/inplasy2021.11.0077 Downloaded from https://inplasy.com/inplasy-2021-11-0077/

depends on the comprehensive analyses of statistical, clinical and methodological heterogeneity. For our meta-analysis, if the statistical heterogeneity is moderate or above by using the fixed-effects model, we will investigate the sources of heterogeneity through subgroup or sensibility analyses for the primary outcome and turn to the random-effects model, because the clinical heterogeneity of acupuncture protocols and settings in included studies is expected. When at least ten studies are included, we will construct funnel plots to assess the likelihood of publication bias.

Subgroup analysis: (1) Type of control: Sham acupuncture, or no adjuvant treatment. (2) Acupuncture sessions: Before, around, or after FET. (3) Total times of acupuncture: Less than 5 times, 5-15 times, or more than 15 times.

Sensitivity analysis: Excluding the studies which are potential contributors to heterogeneity, the meta-analysis will be performed again.

Language: With no restriction.

Country(ies) involved: Mainland China.

Keywords: Infertility; Acupuncture; Frozen-thawed embryo transfer (FET); Pregnancy outcomes; Endometrial morphology.

Contributions of each author:
Author 1 - Can Zhu.
Author 2 - Wanting Xia.
Author 3 - Jinzhu Huang.
Author 4 - Xuan Zhang.
Author 5 - Fangyuan Li.
Author 6 - Xiaorun Yu.
Author 7 - Jiamin Ma.
Author 8 - Qian Zeng.

Zhu et al. Inplasy protocol 2021110077. doi:10.37766/inplasy2021.11.0077 Downloaded from https://inplasy.com/inplasy-2021-11-0077/
