# Supplementary figures and images for: Effects of acupuncture on the pregnancy outcomes of frozen-thawed embryo transfer: A systematic review and meta-analysis
Source: Front Public Health. 2022 Sep 9;10:987276. doi: 10.3389/fpubh.2022.987276 (PMC9501879; doi:10.3389/fpubh.2022.987276)

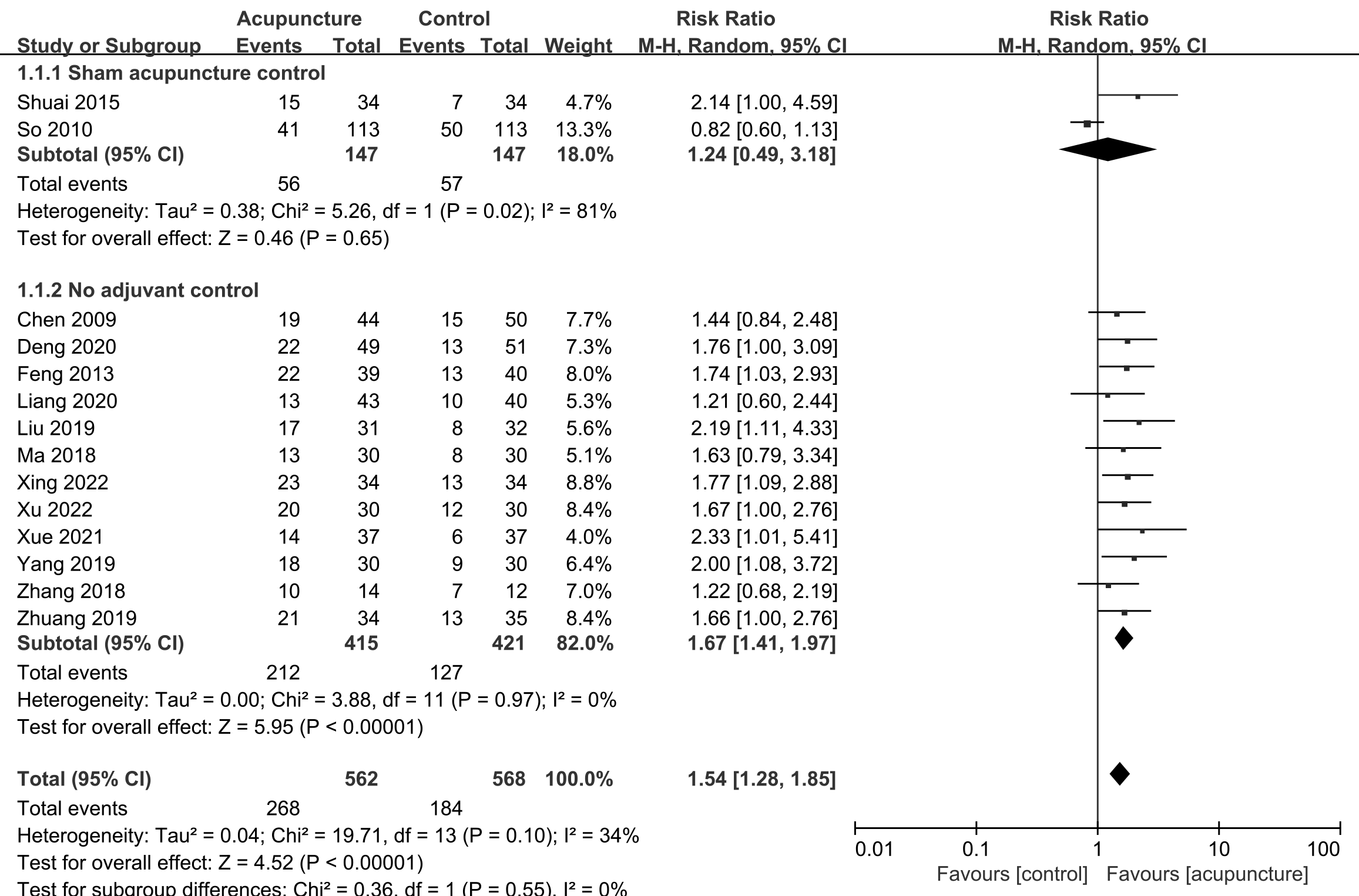

Supplement: Supplementary file 4 [file Image_1.TIF]

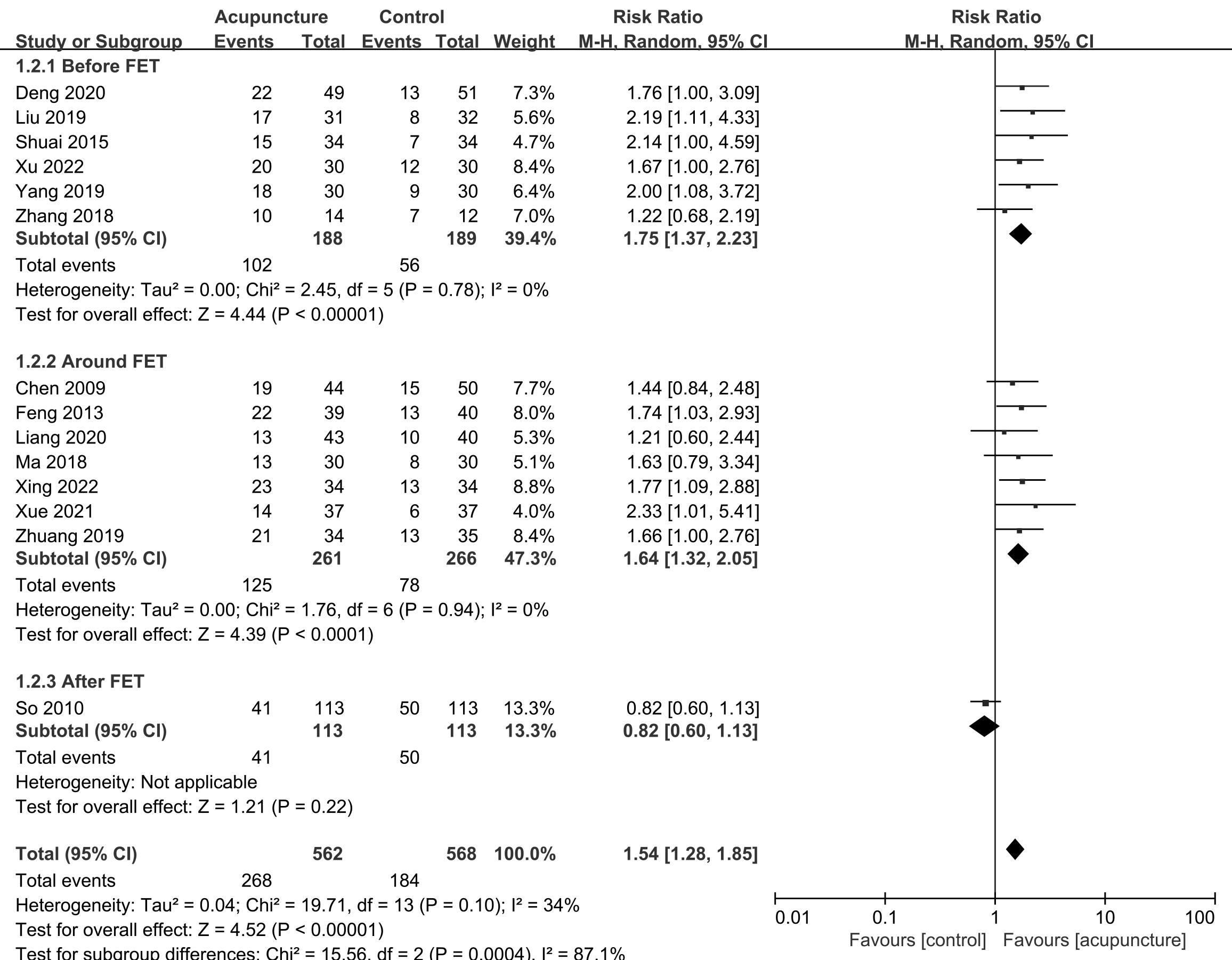

Supplement: Supplementary file 5 [file Image_2.TIF]

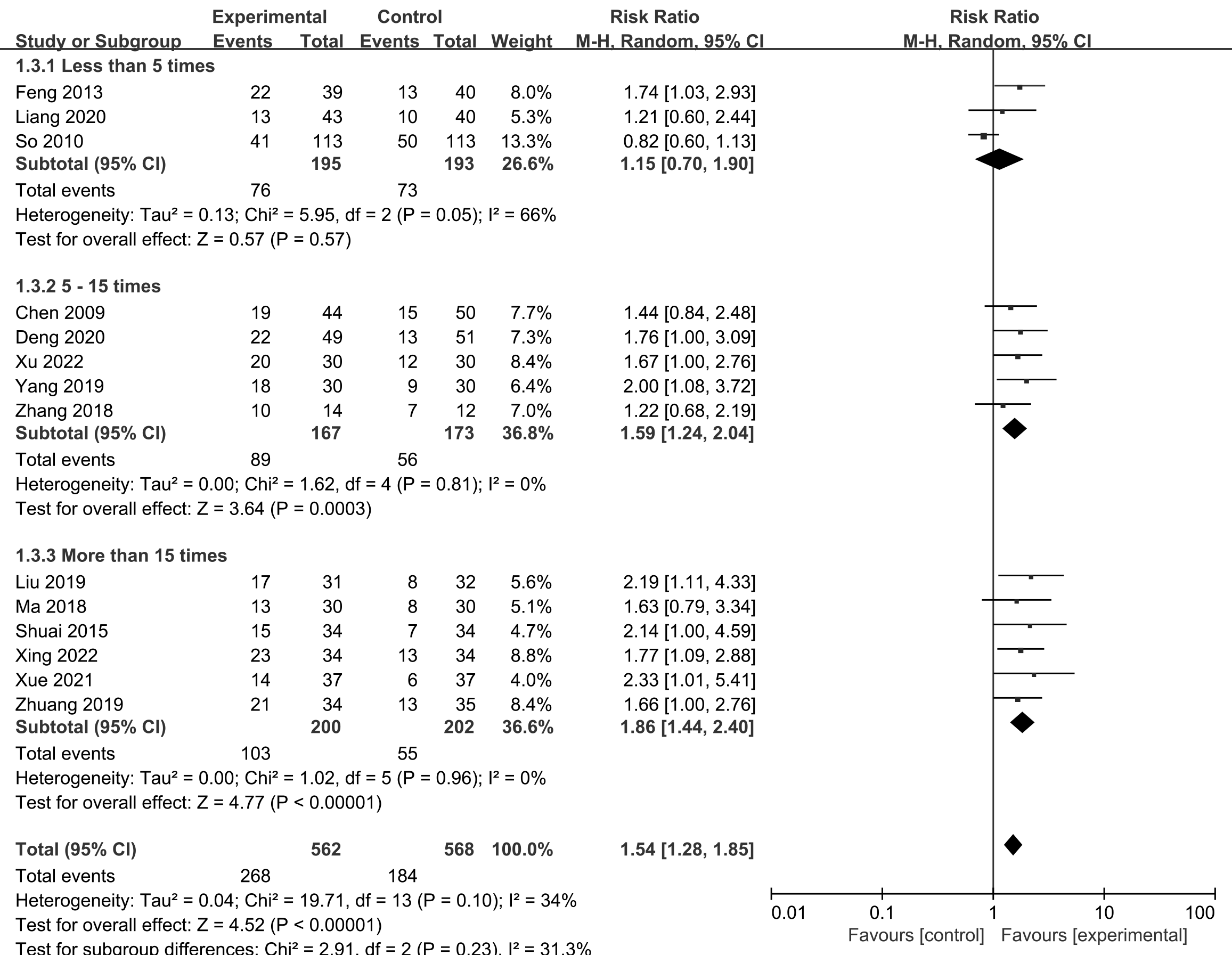

Supplement: Supplementary file 6 [file Image_3.TIF]

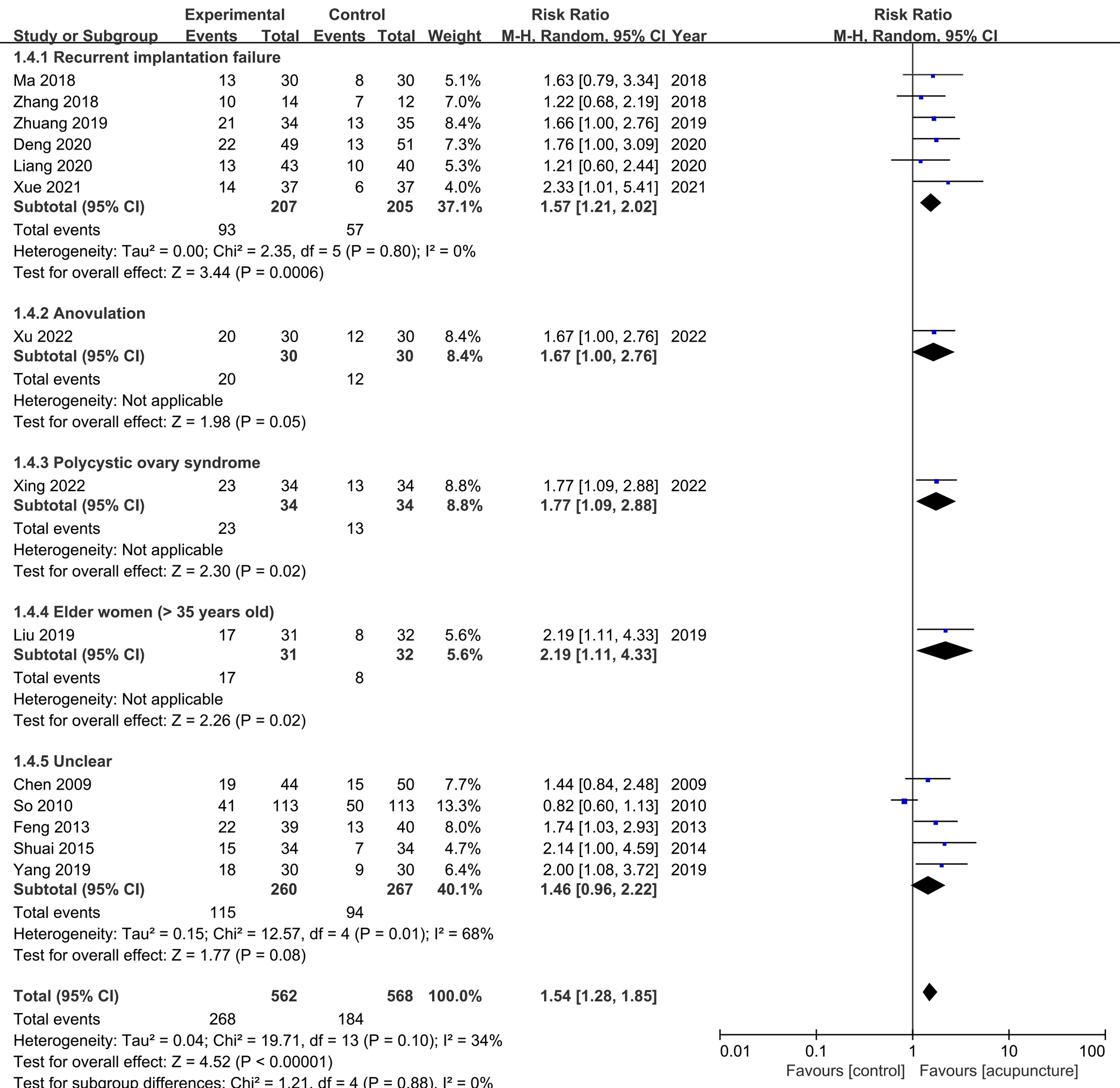

Supplement: Supplementary file 7 [file Image_4.TIF]

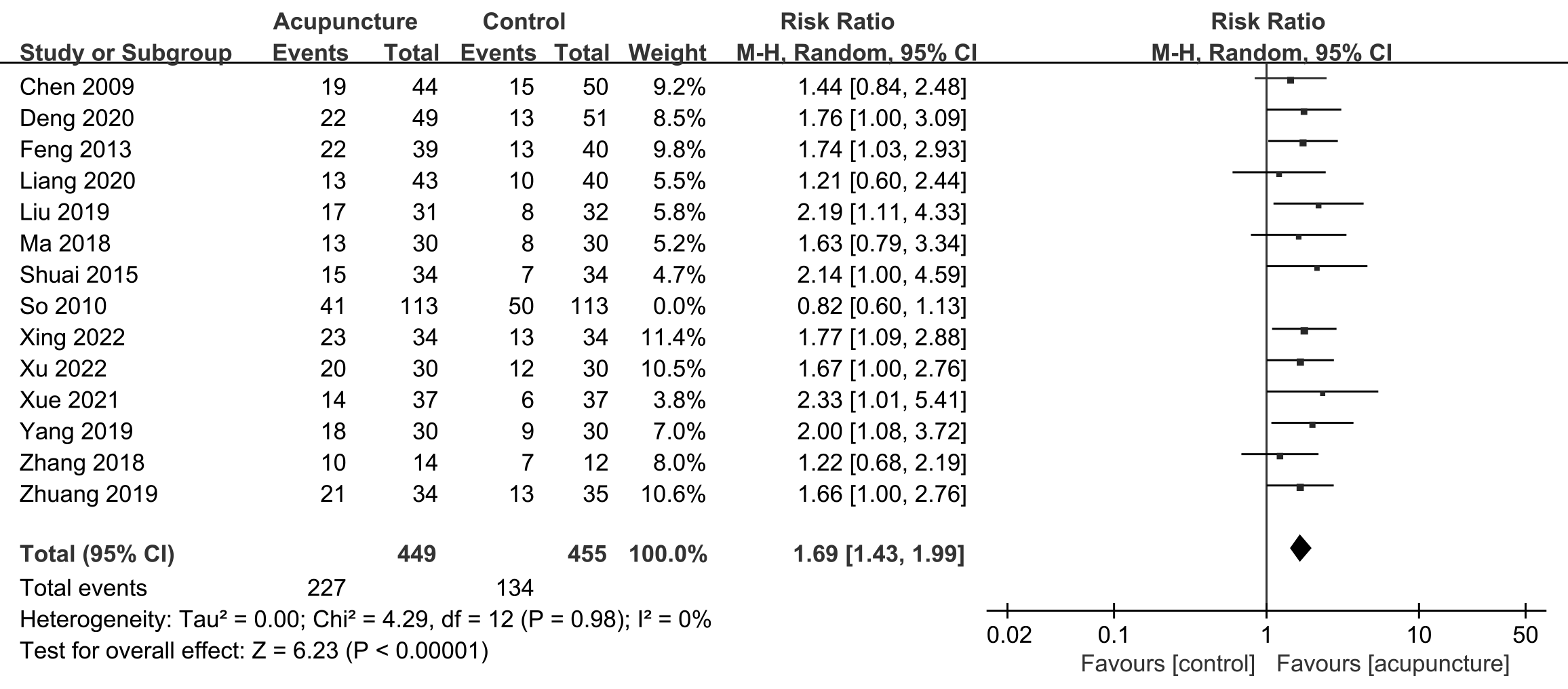

Supplement: Supplementary file 8 [file Image_5.TIF]
